# The Genetic and Epigenetic Features of Bilateral Wilms Tumor Predisposition: A Report from the Children’s Oncology Group AREN18B5-Q Study

**DOI:** 10.21203/rs.3.rs-2675436/v1

**Published:** 2023-03-16

**Authors:** Andrew J. Murphy, Changde Cheng, Justin Williams, Timothy I. Shaw, Emilia M. Pinto, Karissa Dieseldorff-Jones, Jack Brzezinski, Lindsay A. Renfro, Brett Tornwall, Vicki Huff, Andrew L. Hong, Elizabeth A. Mullen, Brian Crompton, Jeffrey S. Dome, Conrad V. Fernandez, James I. Geller, Peter F. Ehrlich, Heather Mulder, Ninad Oak, Jamie Maciezsek, Carolyn Jablonowski, Andrew M. Fleming, Prahalathan Pichavaram, Christopher L. Morton, John Easton, Kim E. Nichols, Michael R. Clay, Teresa Santiago, Jinghui Zhang, Jun Yang, Gerard P. Zambetti, Zhaoming Wang, Andrew M. Davidoff, Xiang Chen

**Affiliations:** St. Jude Children’s Research Hospital; St. Jude Children’s Research Hospital; St. Jude Children’s Research Hospital; St. Jude Children’s Research Hospital; St. Jude Children’s Research Hospital; St. Jude Children’s Research Hospital; The Hospital For Sick Children; Children’s Oncology Group; Children’s Oncology Group; MD Anderson Cancer Center; Emory University; Dana-Farber/Boston Children’s Cancer and Blood Disorders Center; Dana-Farber/Boston Children’s Cancer and Blood Disorders Center; Children’s National Medical Center; Dalhousie University; Cincinnati Children’s Hospital Medical Center; C.S. Mott Children’s Hospital, University of Michigan; St. Jude Children’s Research Hospital; St. Jude Children’s Research Hospital; St. Jude Children’s Research Hospital; St. Jude Children’s Research Hospital; St. Jude Children’s Research Hospital; St. Jude Children’s Research Hospital; St. Jude Children’s Research Hospital; St. Jude Children’s Research Hospital; St. Jude Children’s Research Hospital; University of Colorado Anschutz; St. Jude Children’s Research Hospital; St. Jude Children’s Research Hospital; St. Jude Children’s Research Hospital; St. Jude Children’s Research Hospital; St. Jude Children’s Research Hospital; St. Jude Children’s Research Hospital; St. Jude Children’s Research Hospital

**Keywords:** Bilateral Wilms tumor, 11p15.5, WT1, Wilms tumor predisposition

## Abstract

This study comprehensively evaluated the landscape of genetic and epigenetic events that predispose to synchronous bilateral Wilms tumor (BWT). We performed whole exome or whole genome sequencing, total-strand RNA-seq, and DNA methylation analysis using germline and/or tumor samples from 68 patients with BWT from St. Jude Children’s Research Hospital and the Children’s Oncology Group. We found that 25/61 (41%) of patients evaluated harbored pathogenic or likely pathogenic germline variants, with WT1 (14.8%), *NYNRIN* (6.6%), *TRIM28* (5%) and the BRCA-related genes (5%) *BRCA1, BRCA2,* and *PALB2* being most common. Germline *WT1* variants were strongly associated with somatic paternal uniparental disomy encompassing the 11p15.5 and 11p13/*WT1* loci and subsequent acquired pathogenic *CTNNB1* variants. Somatic coding variants or genome-wide copy number alterations were almost never shared between paired synchronous BWT, suggesting that the acquisition of independent somatic variants leads to tumor formation in the context of germline or early embryonic, post-zygotic initiating events. In contrast, 11p15.5 status (loss of heterozygosity, loss or retention of imprinting) was shared among paired synchronous BWT in all but one case. The predominant molecular events for BWT predisposition include pathogenic germline variants or post-zygotic epigenetic hypermethylation at the 11p15.5 H19/ICR1 locus (loss of imprinting). This study demonstrates that post-zygotic somatic mosaicism for 11p15.5 hypermethylation/loss of imprinting is the single most common initiating molecular event predisposing to BWT. Evidence of somatic mosaicism for 11p15.5 loss of imprinting was detected in leukocytes of a cohort of BWT patients and long-term survivors, but not in unilateral Wilms tumor patients and long-term survivors or controls, further supporting the hypothesis that post-zygotic 11p15.5 alterations occurred in the mesoderm of patients who go on to develop BWT. Due to the preponderance of BWT patients with demonstrable germline or early embryonic tumor predisposition, BWT exhibits a unique biology when compared to unilateral Wilms tumor and therefore warrants continued refinement of its own treatment-relevant biomarkers which in turn may inform directed treatment strategies in the future.

## Introduction

Wilms tumor (WT) is the most common kidney cancer of childhood and 5–7% of WT patients present with synchronous bilateral Wilms tumor (BWT).^1^ BWT development is highly suggestive of an underlying genetic or epigenetic predisposition. In 1972, Knudson and Strong hypothesized that, like retinoblastoma, familial and BWT developed from two genetic events (two-hit hypothesis), the first being either prezygotic (i.e., germline) or postzygotic (i.e., somatic in the early embryo) and the second always postzygotic.^2^ In support of this hypothesis, patients with BWT have a younger median age at diagnosis than those with unilateral WT.^3^ Furthermore, WT precursor lesions known as nephrogenic rests (postnatal persistent clusters of undifferentiated embryonic kidney cells) and multifocal WT are more commonly present in patients with BWT than unilateral WT, supporting the concept of predisposition followed by stepwise accumulation of additional postzygotic somatic events leading to tumor development.^4^

**Figure 1:**
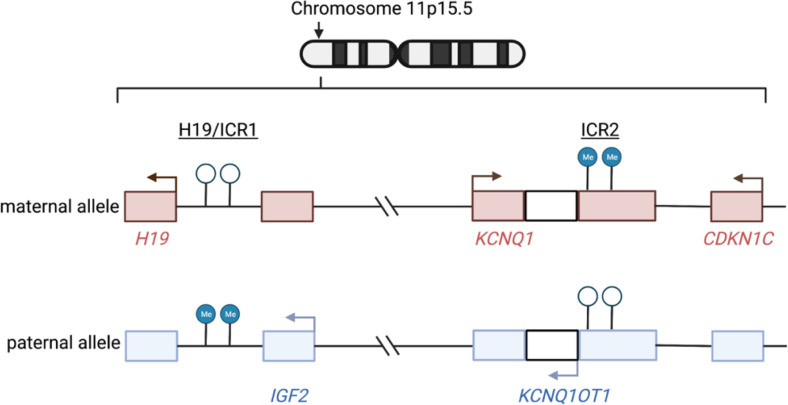
Mechanisms of dysregulated imprinting at chromosome 11p15.5. Chromosome 11p15.5 houses a cluster of imprinted genes (including *IGF2, KCNQ1, CDKN1C*) and noncoding RNAs (including *H19, KCNQ1OT1*) that are either expressed only from the maternal or paternal allele. The expression of these genes is regulated by differential methylation at two imprinting control regions (ICR) – 11p15.5 H19/ICR1 and KCNQ1OT1/ICR2. In normal physiology, H19/ICR1 is methylated on the paternal allele only and *IGF2*is expressed from this allele. In contrast, ICR2, located at the promoter region of *KCNQ1OT1* is methylated on the maternal allele only, leading to expression of *KCNQ1* and *CDKN1C* from the maternal allele. The normal physiologic imprinting status at 11p15.5 is referred to as 11p15.5 retention of imprinting (ROI) throughout this manuscript. Two predominant mechanisms occur that disrupt imprinting and result in biallelic expression of *IGF2*: 1) 11p15.5 H19/ICR1 loss of imprinting (LOI) refers to site-specific epigenetic gain of methylation at H19/ICR1 and 2) 11p15.5 copy neutral loss of heterozygosity (cn-LOH) refers to genetic deletion of the maternal allele and duplication of the paternal allele (paternal uniparental disomy). With 11p15.5 LOI, there is hypermethylation of H19/ICR1 and normal methylation of KCNQ1OT1/ICR2. With 11p15.5 LOH, there is hypermethylation of H19/ICR1 and hypomethylation of KCNQ1OT1/ICR2. Arrows indicate active transcription reflective of the normal physiologic imprinting pattern. Graphic made with biorender.com

When compared with unilateral WT, BWT is more frequent in patients with structural birth defects and known predisposition for WT, including *WT1* disorder (congenital/infantile or childhood onset of steroid-resistant nephrotic syndrome, genitourinary anomalies, predisposition for WT) and WAGR (Wilms tumor, aniridia, genitourinary anomalies, range of developmental delays).^5–10^ In addition, BWT has an increased incidence in patients with Beckwith Wiedemann spectrum disorder (BWSp), implicating dysregulation of imprinting at chromosome 11p15.5, a region which houses a cluster of imprinted genes including the growth factor *IGF2*.^11–13^ The expression of genes and noncoding RNAs at 11p15.5 is controlled by differential methylation of two imprinting control regions (ICR): H19/ICR1 and KCNQ1OT1/ICR2 (Fig. 1). Among patients with BWSp, those with gain of methylation at H19/ICR1 (loss of imprinting) or paternal uniparental disomy (loss of genetic material from the maternal 11p15.5 locus with duplication of the paternal allele in this region; a state known as copy neutral loss of heterozygosity), both of which result in biallelic expression of *IGF2*, have the highest risk for any WT development.^13,14^ The BWSp disease phenotype can vary in severity according to the type of molecular alteration at 11p15.5 and/or mosaic distribution of the alteration throughout the body.^15^ In fact, some patients with mosaic distribution of 11p15.5 abnormalities do not have overt syndromic features and are first diagnosed by detection of subtle clinical abnormalities and germline evaluation at the time of embryonal tumor presentation.^16–19^ For this reason, international consensus guidelines now refer to Beckwith Wiedemann as a spectrum (BWSp) that can be diagnosed using clinical criteria or through molecular testing.^20^

Known genetic variants associated with unilateral WT, including germline pathogenic variants in *WT1* and somatic pathogenic variants in *CTNNB1,* are thought to occur with increased frequency in BWT.^21^ Recent studies have suggested that BWT predisposition in some patients is due to post-zygotic somatic mosaic hypermethylation at H19/ICR1, which results in clonal expansion of histologically normal renal cells (clonal nephrogenesis) and subsequent bilateral and multifocal WT development.^22,23^ However, a comprehensive assessment of the genetic/epigenetic landscape of predisposition for BWT remains undescribed.

The purpose of this study was to determine the landscape of genetic and other molecular events predisposing to BWT using a large cohort of BWT specimens from St. Jude Children’s Research Hospital (SJCRH) and the Children’s Oncology Group (COG). We hypothesized that paired synchronous BWT specimens would exhibit shared genetic or epigenetic predisposing molecular events, while also harboring secondary somatic variants unique to each tumor. Shared genetic or epigenetic events detected in paired synchronous BWT specimens (i.e. in both right and left kidney tumor samples) can be spatially and temporally inferred to occur prior to the lateralization of the mesodermal layer during embryonic gastrulation.^24^ This study provides the first comprehensive assessment of the genetic and epigenetic features of predisposition for BWT, the most common of which is somatic mosaic 11p15.5 H19/ICR1 hypermethylation (loss of imprinting), which is shared among synchronous BWT samples and detectable in the blood cells of a large cohort of BWT patients and long-term survivors. We demonstrate a unique biology for BWT compared to unilateral WT, which warrants continued development of BWT-specific treatment-relevant biomarkers and therapeutic strategies in the future.

## Materials And Methods

### Sample acquisition

This study, including 99 tumor samples from 68 patients with a diagnosis of synchronous BWT (n = 18 SJCRH, n = 50 COG), was approved by the SJCRH institutional review board (IRB# XPD17-052) and approved by the COG as study AREN18B5-Q. Prior to nucleic acid isolation, sections corresponding to all frozen biospecimens were reviewed by a pathologist and determined to have greater than 50% viable tumor. Adjacent non-diseased kidney tissue was confirmed to contain histologically normal kidney and to be tumor-free. We first performed analysis on the discovery cohort of SJCRH BWT specimens from 18 patients. Using preliminary data from this SJCRH discovery analysis, we applied for additional specimens from the COG to establish an expansion cohort. Of note, after determining that somatic variants were almost never shared among synchronous BWT in the SJCRCH cohort using whole exome sequencing, we switched to whole genome sequencing of the COG samples (n = 50 patients) to determine if variants outside coding regions were shared among synchronous BWT.

Genomic DNA and total-RNA were isolated by the SJCRH Biorepository or the COG Biopathology Center. Qiagen DNA extraction kits were used for DNA isolation and Trizol for RNA isolation. DNA was quantified using PicoGreen and visualized in agarose gel for quality control. RNA was quantified using Qubit fluorometry assay and quality and integrity were evaluated using RNA integrity number (RIN) measurements performed using an Agilent Bioanalyzer System.

**Figure 2:**
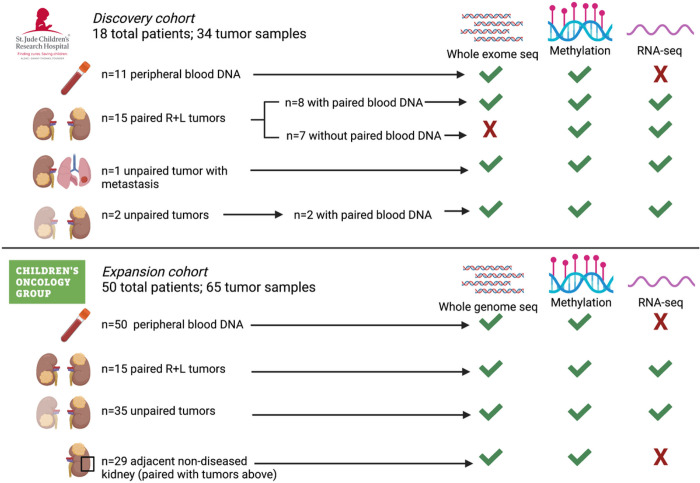
Specimens and molecular assays used in the current study. Whole exome or whole genome sequencing germline variant calls were made using DNA obtained from 61 patients with available DNA from peripheral blood (n=11 SJCRH and n=50 COG). Paired tumor sets are samples from both right and left tumors in a patient with synchronous BWT (n=30). Unpaired tumors are samples from either the right or left tumor in a patient with synchronous BWT, but for whom one side was not available for analysis (n=37). Adjacent non-diseased kidney was confirmed by a pathologist and came from patients with tumors in the study. R – right L – left.

Tumor RNA was used for total strand RNA-seq and tumor DNA for methylation analysis using the 850K methylationEPIC beadchip array (Illumina). Tumor DNA with available matched germline DNA from peripheral blood lymphocytes was used for whole exome (SJCRH) or whole genome sequencing (COG). Germline DNA derived from peripheral blood lymphocytes and adjacent histologically non-diseased kidney was used for whole exome (SJCRH) or whole genome sequencing (COG), and methylation analysis. A detailed account of specimens and sequencing or array modalities utilized in this study is shown in Fig. 2.

### Clinical Data

Clinically relevant details were obtained from each BWT case including patient age at diagnosis, biological sex, tumor laterality, associated congenital anomalies or syndromes, neoadjuvant chemotherapy received, tumor histology, SIOP (Societe Internationale D’oncologie Pediatrique) post-treatment pathology risk strati cation, and presence of nephrogenic rests.^25^

### Whole exome and whole genome sequencing

Whole exome sequencing (SJCRH) or whole genome sequencing (COG) were performed on BWT DNA with available paired germline DNA from peripheral blood (n = 61 patients; n = 87 total tumor samples; Fig. 2). For variant discovery, a paired analysis was performed comparing tumor-derived DNA to germline DNA obtained from peripheral blood leukocytes. Single nucleotide variants, insertion/deletion/frameshift, and noncoding variants calls were made as previously described.^26^ To analyze the pathways affected by somatic variants in BWT we used the functional annotation tool DAVID to generate a set of enriched pathways combining KEGG, Reactome, and Wikipathways.^27^ Using whole exome sequencing or whole genome sequencing data, a copy number variant (CNV) analysis was performed using a threshold of CNV ≥ 0.5 or ≤ −0.5 for full copy number gain or loss at a given chromosomal locus, respectively. Low-level copy number gain or loss was defined as CNV≥ 0.1 and ≤ 0.5 or CNV ≤−0.1 and ≥−0.5 respectively. Areas of copy neutral loss of heterozygosity (cnLOH), loss of heterozygosity (LOH) due to copy loss, or copy number gain were ascertained using the CONSERTING algorithm.^28^

### Total-strand RNA-seq

Total-strand RNA-seq was performed on all BWT samples in the study (n = 99). Total RNA-seq library preparation, sequencing, read mapping, and generation of gene level read counts and Fragments per kilobase million (FPKM) values were generated as previously described.^26^

### Methylation analysis

Genomic tumor and germline DNA were bisulfite converted using the EZ DNA Methylation kit (Zymo Research Corp). Converted samples were processed and hybridized to the Infinium MethylationEPIC Beadchip (850K) array (Illumina) according to the manufacturer’s instructions. Raw IDAT les containing summarized information from the beadchip array were pre-processed using subset-quantile within array normalization (SWAN) function as previously described.^29^ The methylation score of each CpG site in the array is represented as a beta (β) value (methylated signal/methylated + unmethylated signals) and was computed using the R package mini .^30^ Methylation M values (log2 ratio of the intensity of methylated signal/unmethylated signal) were also computed using the R package mini and used for EPIC-based differential methylation analyses including unsupervised hierarchical clustering, TSNE (t-distributed stochastic neighbor embedding), and Spearman correlation matrix analyses.^30,31^

The imprinting status at the chromosome 11p15.5 locus was determined using methylation data as previously described.^32^ Briefly, the average β value for H19/ICR1 was calculated using CpG probes located within the chr11:2,019,974–2,024,738 (GRCh38/hg38) range and the average β value for 11p15.5 KCNQ1OT1/ICR2 was calculated using probes located within the chr11:2,721,228–2,722,228 (GRCh38/hg38) range. Samples with average β value H19/ICR1 < 0.7 and KCNQ1OT1/ICR2 > 0.3 were determined to have normal retention of imprinting (ROI), samples with H19/ICR1 > 0.7 (hypermethylation) and KCNQ1OT1/ICR2 > 0.3 were determined to have loss of imprinting (LOI) at H19/ICR1, and samples with H19/ICR1 > 0.7 (hypermethylation) and KCNQ1OT1/ICR2 < 0.3 (hypomethylation) were determined to have loss of heterozygosity (LOH) at 11p15.5. LOH at 11p15.5 was also designated if samples had LOH or partial LOH detectable using the CONSERTING algorithm from whole genome sequencing data.^28^

### Unsupervised hierarchical clustering, TSNE clustering, Spearman Correlation Matrix

Unsupervised hierarchical clustering was performed using methylation M values from the 850K MethylationEPIC beadchip array data set using all samples from the current BWT data set and BWT and unilateral samples from our prior WT xenograft analysis.^26,31^ The top 10,000 most variable probes in the data set were used for clustering analysis. Probes located on the X and Y chromosomes were excluded to reduce biological sex bias.

### Germline genomic analysis

Germline genetic variants were queried for all patients with an available leukocyte-derived DNA sample from peripheral blood (total n = 61; SJCRH n = 11, COG n = 50). Analysis was performed to query for single nucleotide substitution, nonsense, and insertion/deletion variants in 565 previously described cancer-related genes which specifically include the WT predisposition genes *DICER1, IGF2, TP53, WT1, ASXL1, BRCA2, CDC73, FBXW7, PIK3CA, BLM, BUB1B, CTR9, DIS3L2, GPC3, KDM3B, NYNRIN, PALB2, REST, TRIP13, TRIM28,* and *TRIM37*.^33,34^ All insertion/deletion and nonsense variants were included in germline predisposition variant counts. The Clinvar database (https://www.ncbi.nlm.nih.gov/clinvar/) was queried to determine pathogenicity of single nucleotide variants.^35^ Variants reported as benign in Clinvar were excluded and those reported as pathogenic or probably pathogenic were included in germline predisposition variant counts. Variants of uncertain significance (VUS), reported with no assertion, or unreported variants in Clinvar were further analyzed using the PROVEAN and PolyPhen2 algorithms.^36–38^ Variants classified as deleterious by PROVEAN, possibly damaging or damaging by PolyPhen2 prediction score, were included in germline predisposition variant counts. Furthermore, unreported variants or VUS were included in germline predisposition variant counts if a significant increase in variant allele frequency in the tumor was identified compared to the germline tissue consistent with retention of mutated allele in the tumor (loss of heterozygosity).

### Long-term Survivorship cohort analysis

Blood-derived germline DNA methylation data from the 61 patients in the current study were combined with blood-derived germline DNA methylation data from 282 healthy community controls and 171 long-term Wilms tumor survivors (> 5 years from cancer diagnosis; n = 154 unilateral, n = 17 bilateral) from the St. Jude Lifetime Cohort Study (SJLIFE).^39^ These data were normalized and processed with the subset-quantile within array normalization (SWAN) method using the R package minifi. The average β values from the 11p15.5 H19/ICR1 and KCNQ1OT1/ICR2 regions defined above were computed as detailed above. Using these data, the relationship between age and methylation at 11p15.5 H19/ICR1 was determined using a linear regression model. The relationship between age and methylation from unilateral WT and BWT samples were compared against the learned model from the healthy community control population.

## Results

### Germline variant analysis and associated tumor findings

**Figure 3:**
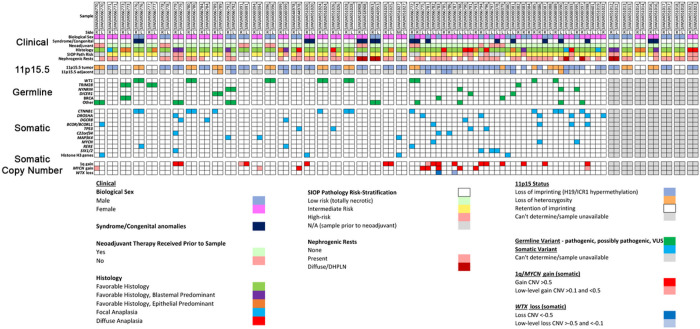
Spectrum of clinical, genetic, and epigenetic features associated with bilateral Wilms tumor. Paired synchronous BWT (n=24) are outlined in paired boxes on the left side of the graphic. Additional paired synchronous BWT (n=7) that did not have a germline peripheral blood sample available are shown in paired boxes on the right side of the graphic. These 7 paired synchronous BWT specimens were analyzed for methylation and RNA-seq only. Gray boxes indicate when a given finding could not be assessed due to sample availability. Unpaired specimens from BWT patients are outlined in the adjacent boxes without spacing in the center of the graphic.

Sixty-one patients (SJCRH n = 11, COG n = 50) with available leukocyte-derived peripheral blood DNA were first assessed focusing on cancer predisposing germline genetic variants. Overall, germline variants in pediatric cancer or WT predisposition genes were found in 25/61 (41%) BWT patients (Fig. 3). Of these 25 patients, 19 had one predisposing germline variant, five had two predisposing germline variants, and one had three predisposing germline variants. Inactivating pathogenic germline variants in *WT1,* a transcription factor critical for normal renal development that has tumor suppressor function in WT^40^, were the most common and found in 9/61 (14.8%) patients. For tumors from patients with germline *WT1* variants, 11p15.5 LOH (paternal uniparental disomy) determined by methylation analysis and/or the CONSERTING algorithm was present in all tumors (Fig. 3). Out of 14 tumor samples from the 9 patients with germline *WT1* variants, 11 (78.6%) were found to have acquired somatic activating variants in exon 3 of *CTNNB1,* which codes for the Wnt pathway effector transcription factor β-Catenin. *CTNNB1* variants were the most common genetic variants in this cohort and converged on Serine at codon 45, a critical residue that is phosphorylated to control nuclear translocation of β-Catenin.^21^ Among 10 total samples harboring germline *WT1* variants, somatic 11p15.5 LOH, and somatic *CTNNB1* variants, there were three sets of paired synchronous BWT (SJWILM066776, 066780, 051028) in which each of the paired tumors had somatic exon 3 *CTNNB1* variants. In two cases, the *CTNNB1* variants were distinct (SJWLM066776 *CTNNB1* p.T41A vs. p.S45del, SJWLM051028 *CTNNB1* p.S45P vs. p.S45del) and in the remaining case (SJWLM066780) the *CTNNB1* p.S45F variant was shared in both paired tumors (Fig. 3).

Germline variants in NYNRIN, a gene for which biallelic truncating variants were previously associated with hereditary WT, were found in 4/61 patients (6.6%).^33^ Germline variants in *TRIM28,* which encodes a nuclear transcriptional co-repressor that coordinates the deposition of repressive histone marks, were found in 3/61 patients (5%), two of whom exhibited epithelial predominant histology in at least one of their tumors, as has been previously shown for germline *TRIM28*-associated WT.^41,42^ All germline *TRIM28*-associated WT were found to have normal chromosome 11p15.5 copy number with ROI. One patient was found to have a *BRCA1* pathogenic missense variant (p.Q687P; SJWLM066773), one patient was found to have a pathogenic frameshift variant (p.T2766fs; SJWLM066783) in *BRCA2,* and one patient was found to have a variant of uncertain significance with predicted deleterious effect by PROVEAN in the BRCA2-related gene *PALB2* (p.S1155C; SJWLM069379). Loss of function mutations in genes coding for the BRCA1-PALB2-BRCA2 protein complex, required for DNA homologous recombination repair, are associated with genomic instability and development of breast and ovarian cancer.^43^ *DICER1* splice site variants of uncertain significance were found in 3 patients (Fig. 3). DICER1 is an endoribonuclease critical for the generation of microRNAs and hereditary mutations in *DICER1* cause the *DICER1* hereditary cancer predisposition syndrome.^44^ Pathogenic somatic variants in microRNA processing genes are commonly found in WT and germline *DICER1* variants have been associated with WT in rare cases.^45,46^ No patient in this cohort was found to have germline numeric or structural alterations on chromosome 11p15.5 at established thresholds for germline 11p15.5 LOI or LOH in leukocyte-derived DNA from peripheral blood; however, mosaic 11p15.5 LOH was identified in the peripheral blood of patient SJWLM069390, who also had frank 11p15.5 LOH detected in an adjacent normal kidney sample and who was reported to have clinical features of BWSp (Supplementary Fig. 1).

The presence of germline variants was strongly associated with the tumor chromosome 11p15.5 status (Chi-square p < 0.0001). Among 37 tumor samples (from 25 patients) with germline variants, 20 had 11p15.5 LOH (54%), 10 had 11p15.5 LOI (27%), and 7 had 11p15.5 ROI (18.9%). Among 52 tumor samples (from 39 patients) with 11p15.5 LOI, 42 did not have germline variants (80.7%) and 10 did have germline variants (19.2%; Fig. 3). These data suggest two groups of predisposing events in BWT: 11p15.5 LOI or a germline genetic variant (often followed by 11p15.5 LOH).

### Somatic variants are not shared in synchronous BWT

**Figure 4:**
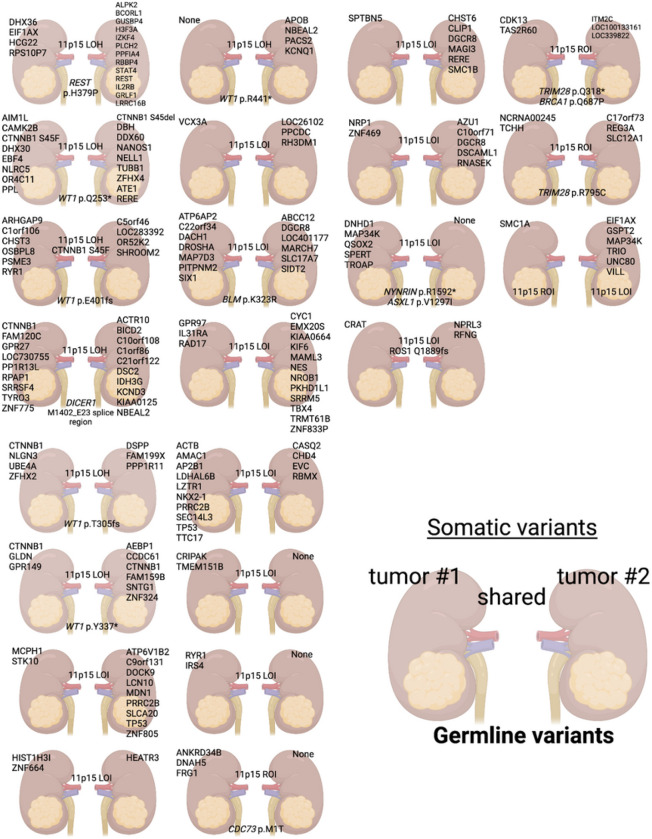
Somatic variants are almost never shared in paired synchronous bilateral Wilms tumor, while chromosome 11p15.5 status is shared in nearly every case. The top 4 rows contain 15 sets of paired synchronous BWT samples obtained from the Children’s Oncology Group and the bottom 4 rows are 8 sets from St. Jude Children’s Research Hospital. Unique/independent somatic variants are indicated over each tumor/kidney. Any shared somatic variants or 11p15.5 status (LOI, LOH, ROI) are indicated between the two kidneys. Any germline variants in pediatric cancer or WT predisposition genes are shown at the bottom of the pair. Graphic made with biorender.com.

The heterogeneous histology and treatment-response (Supplementary Fig. 2) seen in BWT suggest the possibility of differing genetic variants among synchronous tumors in the same patient. Therefore, we conducted a paired analysis of 23 synchronous BWT sets with available matched germline DNA. Among these BWT (SJCRH n = 8; COG n = 15), 21 (91.3%) sample pairs had no shared somatic variants (Fig. 4). For the remaining two synchronous BWT pairs, one pair shared the *CTNNB1* p.S45F (SJWLM066780) and the other the *ROS1* p.Q1889fs variant (SJWLM069391; Fig. 4). Comparison of copy number variants showed markedly different copy number profiles in paired synchronous BWT except for SJWLM069391 (Supplementary Fig. 3–4).

Considering all somatic variants determined by whole genome or whole exome sequencing in 85 available tumor samples, activating *CTNNB1* variants (13/85, 15.3%) were the most common, followed by *DROSHA* (7/85, 8.2%), *BCORL1/BCOR* (5/85, 5.9%), *DGCR8* (4/85, 4.7%), *TP53* (4/85, 4.7%), *SIX1/2* (4/85, 4.7%), *C22orf34* (3/85, 3.5%), *MAP3K4* (3/85, 3.5%), *MYCN* (3/85, 3.5%), and *RERE* (3/85, 3.5%). DAVID pathway analysis revealed that somatic variants in BWT were associated with the RNA/miRNA biogenesis, p53, and generic transcription pathways (Supplementary Table 1).

Regarding copy number variants used for risk stratification in completed or upcoming COG WT protocols, copy number gain at 1q was detected in 17/85 (20%) samples and combined LOH of 1p and 16q was found in 2/85 (2.4%) of samples. 1q gain was shared in 3 of 4 synchronous BWT pairs evaluated. Among these three synchronous BWT pairs with shared 1q gain, two pairs exhibited gain of the entire chromosome 1q arm and (SJWLM069391, SJWLM066779) one pair exhibited a different extent of 1q gain (SJWLM051025; Supplementary Fig. 3). Taken together, these data suggest that 1q gain was likely independent in each tumor rather than from a shared clonal origin (Fig. 3; Supplementary Fig. 3).

Among the COG paired synchronous BWT samples (n = 15 patients, 30 tumors) that underwent whole genome sequencing, we compared shared noncoding somatic variants (Table 1). In this analysis, all paired synchronous BWT without shared noncoding somatic variants (n = 4) had an identifiable germline variant in a WT or pediatric cancer predisposition gene. For all synchronous BWT pairs without an identifiable germline predisposing variant (n = 5), 11p15.5 LOI was detected in addition to shared somatic noncoding variants (Table 1). These data suggest that the primary initiators for BWT predisposition are either germline genetic variants (pre-zygotic), or post-zygotic 11p15.5 LOI that can be inferred to occur early in embryogenesis before the right and left kidney primordia lateralize. This inference is supported by the relatively limited number of shared noncoding somatic mutations in comparison to the overall number of somatic noncoding mutations in each sample (Table 1). Of note, the paired BWT specimens from patient SJWLM069391 were found to have a shared somatic *ROS1* p.Q1889fs variant, 63 shared noncoding mutations, and a nearly identical genome-wide copy number profile, which is atypical among these tumor sets and more consistent with multifocal WT from the same kidney rather than paired BWT.

### 11p15.5 status is shared in synchronous BWT

Thirty paired synchronous BWT (SJCRH = 15, COG = 15) were available for methylation analysis of chromosome 11p15.5 using MethylationEPIC beadchip array data. When available, whole genome sequencing data were also used to detect copy neutral LOH using the CONSERTING algorithm.^28^ Chromosomal 11p15.5 copy number or methylation status (ROI, LOH, LOI; Fig. 1) was shared in 29/30 (96.7%) cases (Supplementary Fig. 5). Overall, of 99 total tumor specimens from BWT patients, 16 (16.1%) had 11p15.5 ROI, 25 (25.2%) had 11p15.5 LOH, and 58 (58.6%) had 11p15.5 LOI for H19/ICR1. No patients were found to meet established thresholds for germline 11p15.5 LOH (H19/ICR1 β > 0.7 and KCNQ1OT1/ICR2 β < 0.3) or LOI (H19/ICR1 β > 0.7 and KCNQ1OT1/ICR2 β > 0.3) in their leukocyte-derived peripheral blood germline DNA sample. However, 9/29 (31%) adjacent non-diseased kidney samples met these thresholds for germline 11p15.5 LOH or LOI (LOH = 2, LOI = 7). When 11p15.5 LOH or LOI was found in adjacent non-diseased kidney tissue, it correlated with the tumor 11p15.5 status in all 9 cases. Among 19 tumors with 11p15.5 LOH determined by whole genome sequencing, the extent of 11p cnLOH overlapped both the 11p15.5 and *WT1*/11p13 loci in 18/19 cases (94.7%; Supplementary Fig. 6). Among paired synchronous BWT from patients with pathogenic germline *WT1* mutations and 11p15.5 LOH detected in each of their tumors, the extent of 11p LOH was not identical. The differential extent of 11p LOH between paired synchronous BWT suggests that 11p LOH occurs as an independent genetic event in each tumor in most cases rather than having a shared clonal origin (Supplementary Fig. 6).

**Figure 5:**
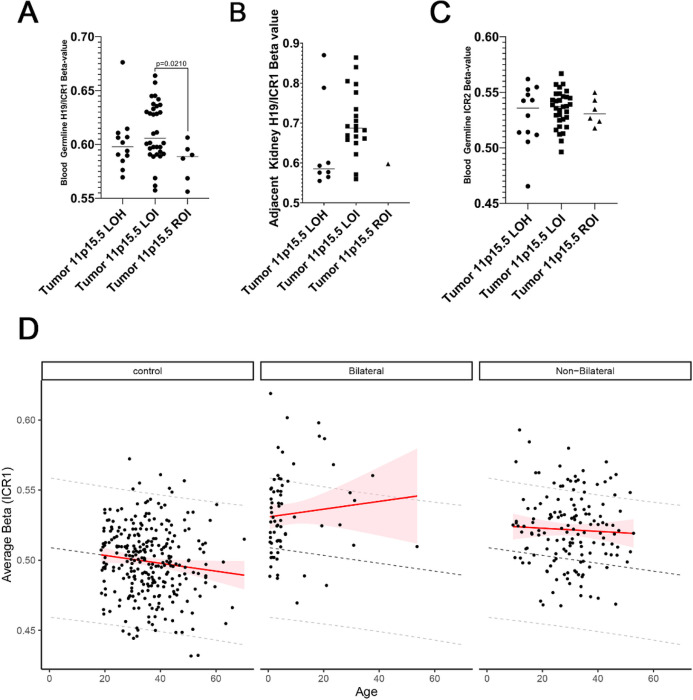
(A) Patients with BWT containing 11p15.5 LOI were found to have a statistically significant increase in 11p15.5 H19/ICR1 methylation detected in the peripheral blood when compared to patients with tumors having 11p15 retention of imprinting (p=0.0210). (B) 11p15.5 LOI was often detectable above the threshold value (b >=0.7) for loss of imprinting in adjacent non-diseased kidney tissues. (C) No differences were detected among patient groups at 11p15.5 KCNQ1OT1/ICR2. Of note, the single sample outlier with tumor 11p15.5 LOH, hypermethylation at H19/ICR1, and hypomethylation at KCNQ1OT1/ICR2detected in the peripheral blood was confirmed to have mosaicism for 11p15 LOH detected in peripheral blood by whole genome sequencing. (D) An increase in H19/ICR1 methylation detected in peripheral blood was noted between BWT patients, BWT long-term survivors, and healthy community controls. 11p15.5 H19/ICR1 methylation increases with age in BWT patients and long-term survivors but decreases with age in healthy community controls and in unilateral WT long-term survivors. Dashed lines demonstrate the predicted change in methylation by age derived from the control group. Shaded red areas indicate the 95% predication interval.

11p15.5 LOI being found in paired synchronous BWT and in adjacent non-diseased kidney tissue (but not above established thresholds in leukocytes) is suggestive of post-zygotic somatic mosaicism. We reasoned that, if present, evidence of post-zygotic somatic mosaicism for chromosome 11p15.5 LOI should be detectable in peripheral blood since hematopoietic progenitor cells have a mesodermal embryonic origin.^47^ To explore whether evidence of somatic mosaicism could be detected in peripheral blood samples from patients with tumors bearing 11p15.5 LOI, we compared the H19/ICR1 β values from leukocyte-derived DNA from peripheral blood among patients according to the 11p15.5 status of their tumors (ROI, LOH, LOI). We found a statistically significant increase in H19/ICR1 methylation detectable in peripheral blood in patients who had tumors with 11p15.5 LOI compared to those with retention of imprinting (Fig. 5). Of note, this statistically significant gain of methylation was low-level with none of the peripheral blood samples achieving the β value of 0.7 associated with frank germline 11p15.5 LOI. In contrast, 7 of the adjacent non-diseased kidney samples met the threshold for frank germline 11p15.5 LOI (Fig. 5).

To validate the finding of somatic mosaicism for 11p15.5 LOI detectable in peripheral blood, we compared H19/ICR1 methylation β values from leukocyte-derived DNA between our BWT cohort and a cohort of healthy community controls and WT cancer long-term survivors (including survivors of both unilateral and BWT) from the St. Jude Life Cohort Study.^39^ In total, this analysis included peripheral-blood derived DNA from 282 healthy community controls, 68 BWT patients and long-term survivors, and 146 unilateral WT patients and long-term survivors. We noted significantly higher levels of H19/ICR1 methylation in BWT patients and long-term survivors as compared to healthy community controls (Fig. 5). We noted an inverse relationship between methylation at H19/ICR1 and increasing age in healthy community controls (Fig. 5). In contrast, we detected positive correlation between increasing age and H19/ICR1 methylation in BWT patients and long-term survivors. This positive correlation was not seen in long-term survivors of unilateral WT (Fig. 5). These results are supportive of 11p15.5 mosaicism as a cause of BWT predisposition.

### 11p15.5 status and genome-wide methylation/molecular identity in BWT

**Figure 6:**
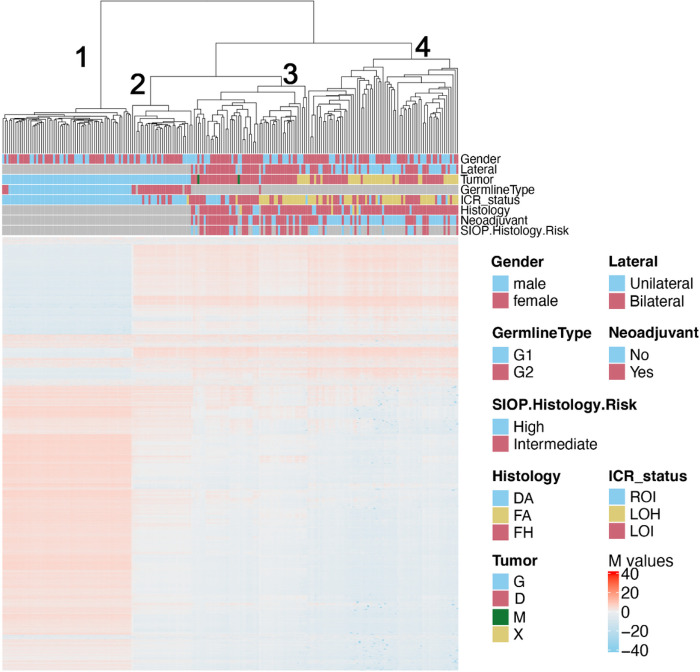
Unsupervised hierarchical clustering of methylation M-values from the top 10,000 most variable probes in the 850K EPIC Methylation Beadchip array demonstrates distinct clustering of DNA samples derived from peripheral blood (cluster 1), adjacent non-diseased kidney (cluster 2), a cluster of predominantly BWT (cluster 3), and a cluster of predominantly unilateral WT (cluster 4). Within the BWT cluster (cluster 3), samples with 11p15.5 LOH, 11p15.5 LOI, and 11p15.5 ROI cluster together. Notably, the BWT cluster (cluster 3) joins with adjacent non-diseased kidney (cluster 2) more closely than the unilateral WT cluster. Germline type: G1 – blood, G2 – kidney. Histology – DA – diffuse anaplasia, FA – focal anaplasia, FH – favorable histology. Tumor type: G – germline sample, D – primary tumor sample, M – metastatic sample, X – patient-derived xenograft.

**Figure 7:**
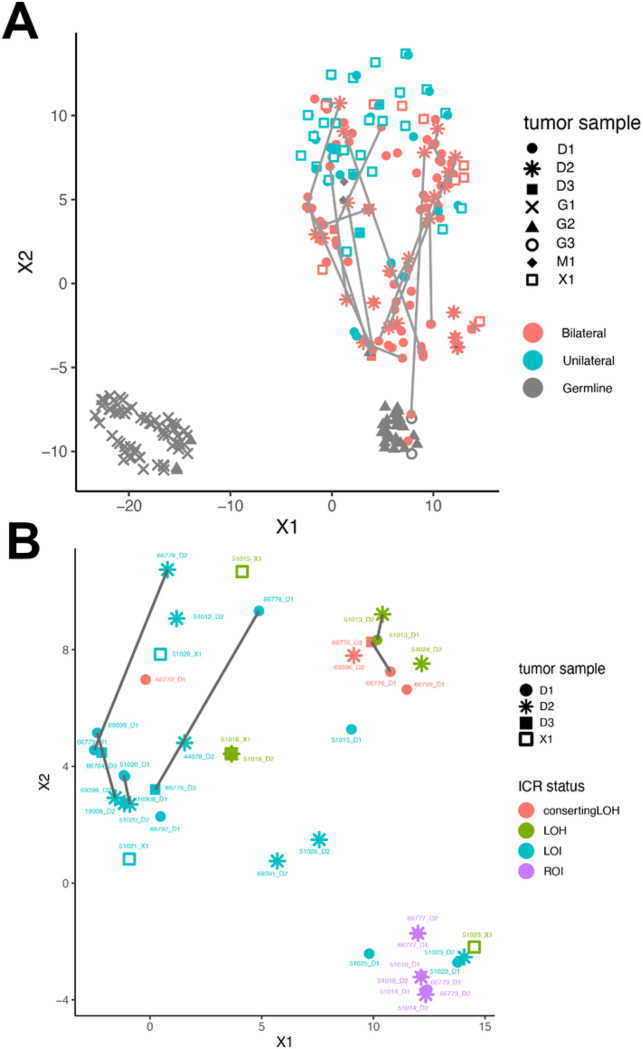
TSNE clustering analysis of 850K EPIC Methylation Beadchip array. (A) Predominant clusters of unilateral (teal) and bilateral (salmon) WT. BWT cluster closer to adjacent non-diseased kidney (gray triangles) than unilateral WT. Gray lines connect synchronous BWT. However, large differences in synchronous BWT correlated with differential tumor purity in each DNA sample. (B) Clustering of BWT with a tumor purity filter applied (excluding specimens with tumor purity < 80%) shows near adjacent clustering of paired synchronous BWT specimens. Within BWT, samples cluster according to 11p15.5 status: 11p15.5 LOI (light blue), 11p15.5 LOH (red and green), and 11p15.5 ROI (purple). The greatest difference between paired synchronous BWT samples is seen in samples with 11p15.5 LOI (samples connected by dashed lines).

We performed unsupervised hierarchical clustering using methylation M values from the top 10,000 most variable probes in the 850K methylationEPIC dataset from leukocyte-derived DNA, adjacent non-diseased kidney DNA, BWT specimens, and unilateral WT specimens. To include unilateral WT specimens in this analysis for comparison, we combined the data set from the current study with methylation data from our previous analysis of WT primary tumors and corresponding xenografts.^26^ We noted that BWT predominantly clustered distinctly from unilateral WT and closer to non-diseased adjacent kidney tissue, consistent with previously published results (Fig. 6).^48^ Within the BWT cluster, subgroups of tumors found to have 11p15.5 LOH, LOI, and ROI clustered together. To validate this result using a different computational method, we performed TSNE clustering of 850K methylation EPIC data, which also showed BWT clustered distinctly from unilateral WT and closer to adjacent non-diseased kidney tissue (Fig. 6). Among BWT, clustered subgroups of 11p15.5 LOH, LOI, and ROI emerged in TSNE analysis. Paired synchronous BWT with 11p15.5 LOI had the greatest inter-tumor variability in clustering pattern, and this result was confirmed using a Spearman correlation matrix (Fig. 7, Supplementary Fig. 7). These data suggest that 11p15.5 status serves as a biomarker for global methylation/molecular subgroups in BWT and that BWT have distinct methylation patterns from unilateral WT, with BWT being more similar in global methylation to non-diseased kidney tissue.

## Discussion

**Figure 8:**
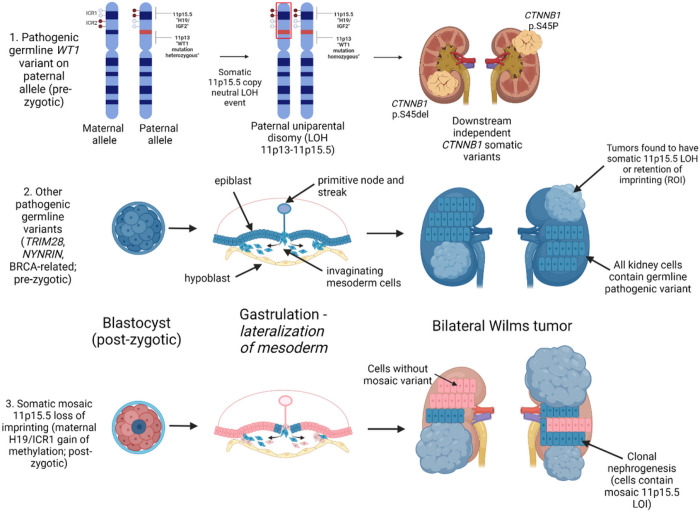
Summary of Proposed Mechanisms for Bilateral Wilms tumor development. The top panel depicts the molecular sequence leading to BWT in patients with pathogenic heterozygous *WT1* germline variants. Here, somatic 11p15.5 copy neutral loss of heterozygosity causes biallelic inactivation of *WT1* and biallelic expression of IGF2, a sequence which is often followed by downstream distinct *CTNNB1* somatic variants unique to each tumor. The middle panel depicts the general sequence of BWT development due to a pre-zygotic pathogenic germline variant in which the germline variant is present in all kidney cells. The bottom panel depicts somatic mosaic 11p15.5 loss of imprinting, in which 11p15.5 H19/ICR1 gain of methylation occurs on the maternal allele in a post-zygotic embryonic cell. This event must occur prior to lateralization of cells fated to become mesoderm during embryonic gastrulation. At this time in embryonic development, the cells that give rise to the intermediate mesoderm and therefore the kidneys are anatomically sequestered from one another (right and left). This lateralization results in a somatic mosaic distribution of 11p15.5 LOI throughout the body/mesoderm. Expansion of cellular clones containing the somatic mosaic alteration is termed clonal nephrogenesis and explains the detection of 11p15.5 LOI in adjacent non-diseased kidney tissue. BWT and/or multifocal WT arise from these clonal populations of kidney cells. Graphic made with biorender.com

This study determined the landscape of predisposition for bilateral Wilms tumor. These data demonstrate two predominant modes of BWT susceptibility that both support the Knudson and Strong two-hit hypothesis: 1) Pre-zygotic germline genetic variants (*WT1, NYNRIN, TRIM28,* BRCA complex genes) or 2) Post-zygotic epigenetic hypermethylation at the 11p15.5 H19/ICR1 locus (loss of imprinting; Fig. 8). The 11p15.5 H19/ICR1 gain of methylation is shared in paired synchronous BWT and can be inferred to occur in the early embryo given the small number of shared noncoding variants relative to the total number of noncoding variants demonstrated in each paired tumor. This inference can also be made because the mesodermal cells that give rise to the right and left intermediate mesoderm/kidney primordia are spatially isolated after the period of embryonic gastrulation when they invaginate and migrate to the right or left lateral sides of the embryo (Fig. 8). Additional support for 11p15.5 H19/ICR1 hypermethylation being a post-zygotic, somatic mosaic event include: (1) the frequent detection of 11p15.5 H19/ICR1 hypermethylation at frank germline thresholds (β > 0.7) in adjacent non-diseased kidney tissue and associated tumors but not blood, (2) the statistically significant increase H19/ICR1 methylation detected in peripheral blood in patients with 11p15.5 H19/ICR1 hypermethylation detected in their tumors, and (3) the positive correlation between 11p15.5 H19/ICR1 methylation and age in patients and long-term survivors with BWT, but not unilateral WT or in healthy community controls. Therefore, we found BWT predisposition in the absence of a predisposing germline variant is a manifestation of the Beckwith Wiedemann spectrum often without overt additional clinical features.

*WT1* variants are the most common germline genetic variants associated with BWT predisposition and were found in 9/61 (14.8%) patients who underwent peripheral blood germline genetic sequencing in this cohort. In contrast to *WT1* variants of purely somatic origin, which are often seen in unilateral WT, all patients with tumor *WT1* alterations in the current study were found to have a germline variant in *WT1*.^21^ Scott *et al.* and Huff *et al.* similarly found germline *WT1* variants in BWT specimens containing *WT1* alterations.^49,50^ These data demonstrate a strong association between *WT1* germline variants and somatic 11p15.5 copy neutral LOH events (paternal uniparental disomy) that encompass both the 11p15.5 and *WT1*/11p13 loci, resulting in biallelic expression of *IGF2* and biallelic inactivation of *WT1* (Fig. 8). Therefore, it can be inferred that the germline genetic variants that lead to BWT predisposition occur on the paternal allele and become homozygous in the tumor due to copy neutral LOH events at 11p13-11p15.5 loci that establish paternal uniparental disomy. The exact breakpoints of 11p15.5 LOH are different in paired synchronous BWT, demonstrating convergent tumor evolution via independent genetic events in each tumor specimen, consistent with the study by Valind et. al.^51^ This sequence is often followed by development of activating somatic *CTNNB1* variants, which were the most common somatic variants found in the current study. Our results are consistent with the temporal sequence first reported by Fukuzawa et. al. who found that *WT1* variants were found in nephrogenic rests and WT, but that accompanied *CTNNB1* variants were only found in WT.^52^ *WT1* and *CTNNB1* variants often co-occur in WT and the spatial distribution of *CTNNB1* variants has been demonstrated to exhibit intra-tumor genetic heterogeneity.^21,53^ WT driven by *WT1* variants are known to exhibit stromal and rhabdomyoblastic differentiation to neoadjuvant chemotherapy, which may result in poor volumetric regression.^54,55^ Therefore, future knowledge of germline *WT1* status at diagnosis in patients with BWT could guide expectations regarding volumetric tumor regression and timing of surgical resection. Taken together, these data and a recent study by Hol. et. al that demonstrated a much higher than predicted incidence of germline *WT1* variants in unselected WT patients (either unilateral or bilateral, often without syndromic features), support expanded germline genetic testing at diagnosis for all patients with BWT.^19^

Tumors with germline genetic variants in pediatric cancer or WT predisposition genes in this study were most often found to exhibit 11p15.5 loss of heterozygosity (with different regions of LOH in paired synchronous BWT) or to maintain normal physiologic 11p15.5 retention of imprinting. Both scenarios suggest the germline genetic variant as the primary cause of BWT predisposition and 11p15.5 LOH (when present) as a somatic secondary event contributing to tumorigenesis. In contrast, tumors with 11p15.5 H19/ICR1 hypermethylation (LOI) were frequently found without associated germline genetic variants and in conjunction with a wide variety of somatic variants different in each paired BWT specimen. These findings suggest 11p15.5 LOI itself is the primary cause for BWT predisposition in the absence of a germline predisposing variant, a condition which was found in 31/61 (51%) patients evaluated by germline genetic sequencing in this study. Consistent with this possibility, frank 11p15.5 LOI (H19/ICR1 ß > 0.7) was found in adjacent histologically non-diseased kidney tissue in 7 of 29 (24%) evaluated specimens. 11p15.5 LOH was found in 2/29 (6.9%) adjacent histologically non-diseased kidney samples, indicating that this finding precedes tumor development in a small number of cases. In one of these two cases (SJWLM069390, patient with clinical diagnosis of BWSp), germline mosaicism for 11p15.5 LOH was confirmed in peripheral blood using whole genome sequencing data, demonstrating that 11p15.5 LOH can also occur as a mosaic phenomenon at a lower frequency in BWT patients than 11p15.5 LOI. Chao et. al previously demonstrated mosaicism for 11p15 LOH in normal tissues (including peripheral blood) of 4 WT patients with varying degrees of BWSp phenotypes, but showed that the degree of partial LOH detected in the blood was less than that in adjacent non-diseased kidney.^18^

Okamoto et. al. originally described mosaicism for 11p15.5 LOI in mesodermal tissues of patients with WT, including adjacent non-diseased kidney tissue of 8/8 patients with tumor 11p15.5 LOI in their study.^56^ In addition one of these eight patients (who had a clinically apparent BWSp phenotype) was found to have increased H19 methylation detectable in peripheral blood.^56^ Our current results build on these findings and the recent detailed analyses performed on a focused set of BWT specimens by Coorens et. al because we establish the relative frequency of 11p15.5 LOI somatic mosaicism in the overall landscape of BWT predisposition and provide evidence that this finding can be detected in the peripheral blood, thereby establishing a mechanism more consistent with mosaicism throughout tissues of the mesoderm rather than limited to the kidney.^22^ Coorens et. al showed that 11p15.5 LOI likely occurs as a post-zygotic event that results in somatic mosaicism for H19/ICR1 hypermethylation. Like our results, their analysis showed shared noncoding variants and associated hypermethylation of ICR1/H19 in specimens from two BWT patients. Also in their study, the H19/ICR1 hypermethylation was detected in adjacent non-diseased kidney tissue and associated with clonal expansion of histologically normal renal cells deemed “clonal nephrogenesis.” This clonal expansion of nephrogenic cells provides the precursor cell population for multifocal and BWT development. Our detection of 11p15.5 LOI or LOH in 9/29 (31%) samples of adjacent histologically non-diseased kidney tissue is also consistent with the concept of clonal nephrogenesis. Although Coorens *et al.* concluded that the mosaic distribution results in H19/ICR1 hypermethylation detectable in adjacent non-diseased kidney, but not in peripheral blood, we reasoned that because lymphocytes are derived from the embryonic mesodermal cell population that there should be some evidence of somatic mosaicism detected in peripheral blood samples. In addition, we found a single case of somatic mosaicism for 11p15.5 LOH ascertained by whole genome sequencing in this study.

Indeed, our analysis showed a statistically significant increase in H19/ICR1 methylation in peripheral blood from patients with 11p15.5 LOI in their tumor specimens when compared to those with 11p15.5 retention of imprinting in their tumors, further supporting the concept of epigenetic somatic mosaicism with detection of low-level gain of methylation. We validated this observation by comparing H19/ICR1 peripheral blood methylation levels in our BWT cohort to a cohort of healthy community controls and unilateral WT long-term survivors. We found a positive correlation between increased age and H19/ICR1 methylation that was specific to BWT patients and long-term survivors and not found in healthy community controls or unilateral WT patients or long-term survivors. We speculate that increased methylation with age at 11p15.5 H19/ICR1 in the BWT population could be due to gradual selection/expansion of cellular clones with H19/ICR1 hypermethylation in the peripheral blood over time in a manner similar to what was described as clonal nephrogenesis in the kidney.^22^ Progressive selection of mosaic hematopoetic cells containing uniparental paternal isodisomy for 11p15 has been suggested as a mechanism for late onset β-thalassemia major in patients who are heterozygous carriers of pathogenic *HBB* variants (which is also located at 11p15).^57^ These results are very unlikely to be related to circulating tumor DNA because the positive correlation between age and H19/ICR1 methylation was also seen in BWT long-term survivors, whose DNA samples were obtained at least 5 years after cancer diagnosis. These results are consistent with the single-institution study by Fiala *et al.* who detected low-level gain of methylation (which they defined as methylation greater than two standard deviations above normal) at H19/ICR1 in 8 of 24 (33%) patients assessed with WT or hepatoblastoma, a phenomenon that was seen recurrently in females with BWT in their study.^23^

Furthermore, our data show that 11p15.5 status can be used as a surrogate biomarker for more global methylation patterns/molecular subgroups of BWT. Whether 11p15.5 status (ROI, LOI, LOH) correlates with volumetric or histologic response to neoadjuvant chemotherapy, event-free, or overall survival will be the subject of future clinical translational investigation. However, as a preliminary window into this question, the current study showed that 15/17 (88.2%) specimens with SIOP high-risk post-treatment histology and 14/17 (82.4%) with the adverse prognostic biomarker 1q gain had 11p15.5 LOI. Chromosome 11p15.5 LOH or LOI were associated with disease relapse in very-low risk (Stage I, < 2 years of age, tumor weight < 500 g) unilateral WT patients treated with surgery alone and no chemotherapy or radiation.^58^ However, 11p15.5 status does not correlate with outcome in unselected, predominantly unilateral WT. The current study provides strong rationale for prospectively following outcomes in BWT patients according to tumor 11p15.5 status and this will be included as an observational biologic aim in the Children’s Oncology Group BWT protocol currently under development.

This study has limitations. The study was designed to maximize the number of eligible paired synchronous BWT specimens. Thus, not all tumors were treated according to the same protocol, some tumors samples were obtained as pre-treatment biopsies, and some samples were obtained after neoadjuvant therapy was administered at the time of surgical resection. Still, the number of paired synchronous BWT specimens was limited by inconsistent sample collection, insufficient tumor purity, availability, and heterogeneous treatment response which could have caused some tumors to be ineligible for inclusion due to necrosis, stroma, or tumor content thresholds. Furthermore, the study does not account for intra-tumor genetic heterogeneity known to be present in WT since only a single sample was included from each tumor. Therefore, prospective collection of BWT samples treated under a uniform protocol will be needed for clinical translational aims such as the association between biologic subgroups and treatment response to neoadjuvant therapy or long-term oncologic outcomes.

In conclusion, contemporary molecular diagnostics have enabled us to provide strong support for the Knudson and Strong two-hit hypothesis of BWT predisposition. Our study shows that the predisposition for BWT occurs primarily due to pre-zygotic germline genetic variants or post-zygotic 11p15.5 loss of imprinting (H19/ICR1 hypermethylation). These findings underscore the rationale for an (epi)genotype first approach in which molecular diagnostics can be used to subgroup BWT patients according to germline variants and/or 11p15.5 copy number and/or epigenetic alterations to determine how these modes of predisposition correlate with volumetric or histologic tumor response and long-term oncologic outcomes. These findings also suggest that in-depth (epi)genetic testing including genetic sequencing and methylation analysis of peripheral blood, adjacent kidney (when available), and tumor may be required to diagnose the means of predisposition to BWT. The determination of BWT predisposition due to germline genetic variants would warrant consideration of genetic testing for family members. In contrast, BWT predisposition because of somatic mosaic 11p15.5 LOI is a post-zygotic event and would therefore not warrant familial testing. The method of BWT predisposition can be ascertained in most cases with appropriate tissue resources and testing. Detection of 11p15.5 LOI as a means of predisposition may warrant additional tumor screening according to BWSp management guidelines.^59^ The frequency of inherited versus *de novo* BWT predisposing germline variants is also a subject worthy of future investigation and will require analysis of parental DNA. Because BWT is driven by germline or early embryonic predisposition, its biology is unique from unilateral WT and therefore future treatment strategies and clinical translational biomarkers may need to be considered separately from unilateral WT.

## Figures and Tables

**Table 1.** Shared noncoding variants in paired synchronous bilateral Wilms tumor samples that underwent whole genome sequencing (n=15). LOH – loss of heterozygosity, LOI – loss of imprinting, ROI – retention of imprinting.

Case ID	Tumor 1 Noncoding Somatic Variant Counts	Tumor 2 Noncoding Somatic Variant Counts	Shared Noncoding Somatic Variant Counts	11p15.5 Status	Predisposing Germline Variants
SJWLM066770	136	119	0	LOH	*REST*
SJWLM066771	54	74	6	LOI	No
SJWLM066773	38	41	0	ROI	*TRIM28, BRCA1*
SJWLM066776	122	174	5	LOH	*WT1*
SJWLM066777	69	59	0	ROI	*TRIM28*
SJWLM066778	379	68	1	LOI	No
SJWLM066779	83	230	0	LOI	*BLM*
SJWLM066780	97	104	0	LOH	*WT1*
SJWLM066784	73	83	1	LOI	No
SJWLM066789	125	58	2	LOH	*DICER1*
SJWLM066792	79	16	1	LOI	*ASXL1, NYNRIN*
SJWLM069391	102	121	63	LOI	No
SJWLM069394	43	104	1	LOI/ROI	No
SJWLM069396	38	90	2	LOH	*WT1*
SJWLM069399	136	55	1	LOI	No

## Data Availability

850K MethylationEPIC data from the St. Jude and Children’s Oncology Group bilateral Wilms tumor patients in the current study are uploaded to the Gene Expression Omnibus (GEO) database (https://www.ncbi.nlm.nih.gov/geo/) under accession number GSE226234. RNA-seq data are available in the GEO database under accession number GSE226480, Whole exome sequencing, and whole genome sequencing data from this cohort are being uploaded to the NCBI Sequence Read Archive (SRA) database currently and accession numbers will be provided when assigned. 850K MethylationEPIC data used from our prior publication (Murphy, et. al. Nat Commun. 2019 Dec20;10(1):5806. doi: 10.1038/s41467-019-13646-9) which are used for comparative analysis in the current study are available via the GEO database under accession number GSE110697. 850K MethylationEPIC data from the Wilms tumor survivorship cohort and healthy community controls (Song, et. al. Genome Med. 2021 Apr 6;13(1):53. doi: 10.1186/s13073-021-00875-1) are available via the GEO database under accession numbers GSE197676, GSE197675, and GSE197674.

## References

[1] Davidoff A.M. Wilms tumor. Adv Pediatr 59, 247–67 (2012). 22789581 10.1016/j.yapd.2012.04.001 PMC3589819

[2] Knudson A.G. & Strong L.C. Mutation and cancer: a model for Wilms’ tumor of the kidney. J Natl Cancer Inst 48, 313–24 (1972). 4347033

[3] Spreafico F. Wilms tumour. Nat Rev Dis Primers 7, 75 (2021). 34650095 10.1038/s41572-021-00308-8

[4] Beckwith J.B., Kiviat N.B. & Bonadio J.F. Nephrogenic rests, nephroblastomatosis, and the pathogenesis of Wilms’ tumor. Pediatr Pathol 10, 1–36 (1990). 2156243 10.3109/15513819009067094

[5] Chernin G. Genotype/phenotype correlation in nephrotic syndrome caused by WT1 mutations. Clin J Am Soc Nephrol 5, 1655–62 (2010). 20595692 10.2215/CJN.09351209 PMC2974408

[6] Lehnhardt A. Clinical and molecular characterization of patients with heterozygous mutations in wilms tumor suppressor gene 1. Clin J Am Soc Nephrol 10, 825–31 (2015). 25818337 10.2215/CJN.10141014 PMC4422247

[7] Lipska B.S. Genotype-phenotype associations in WT1 glomerulopathy. Kidney Int 85, 1169–78 (2014). 24402088 10.1038/ki.2013.519

[8] Royer-Pokora B. Twenty-four new cases of WT1 germline mutations and review of the literature: genotype/phenotype correlations for Wilms tumor development. Am J Med Genet A 127A, 249–57 (2004). 15150775 10.1002/ajmg.a.30015

[9] Hol J.A. Wilms tumour surveillance in at-risk children: Literature review and recommendations from the SIOP-Europe Host Genome Working Group and SIOP Renal Tumour Study Group. Eur J Cancer 153, 51–63 (2021). 34134020 10.1016/j.ejca.2021.05.014

[10] Hol J.A. Clinical characteristics and outcomes of children with WAGR syndrome and Wilms tumor and/or nephroblastomatosis: The 30-year SIOP-RTSG experience. Cancer 127, 628–638 (2021). 33146894 10.1002/cncr.33304 PMC7894534

[11] Ehrlich P. Results of the First Prospective Multi-institutional Treatment Study in Children With Bilateral Wilms Tumor (AREN0534): A Report From the Children’s Oncology Group. Ann Surg 266, 470–478 (2017). 28795993 10.1097/SLA.0000000000002356 PMC5629006

[12] Ehrlich P.F. Results of Treatment for Patients With Multicentric or Bilaterally Predisposed Unilateral Wilms Tumor (AREN0534): A report from the Children’s Oncology Group. Cancer 126, 3516–3525 (2020). 32459384 10.1002/cncr.32958 PMC7769115

[13] Brzezinski J. Wilms tumour in Beckwith-Wiedemann Syndrome and loss of methylation at imprinting centre 2: revisiting tumour surveillance guidelines. Eur J Hum Genet 25, 1031–1039 (2017). 28699632 10.1038/ejhg.2017.102 PMC5558170

[14] Weksberg R. Tumor development in the Beckwith-Wiedemann syndrome is associated with a variety of constitutional molecular 11p15 alterations including imprinting defects of KCNQ1OT1. Hum Mol Genet 10, 2989–3000 (2001). 11751681 10.1093/hmg/10.26.2989

[15] Slatter R.E. Mosaic uniparental disomy in Beckwith-Wiedemann syndrome. J Med Genet 31, 749–53 (1994). 7837249 10.1136/jmg.31.10.749 PMC1050119

[16] MacFarland S.P. Diagnosis of Beckwith-Wiedemann syndrome in children presenting with Wilms tumor. Pediatr Blood Cancer 65, e27296 (2018). 29932284 10.1002/pbc.27296 PMC6107414

[17] Scott R.H. Constitutional 11p15 abnormalities, including heritable imprinting center mutations, cause nonsyndromic Wilms tumor. Nat Genet 40, 1329–34 (2008). 18836444 10.1038/ng.243

[18] Chao L.Y. Genetic mosaicism in normal tissues of Wilms’ tumour patients. Nat Genet 3, 127–31 (1993). 8388768 10.1038/ng0293-127

[19] Hol J.A. Prevalence of (Epi)genetic Predisposing Factors in a 5-Year Unselected National Wilms Tumor Cohort: A Comprehensive Clinical and Genomic Characterization. J Clin Oncol, JCO2102510 (2022). 10.1200/JCO.21.02510 PMC9177240 35230882

[20] Brioude F. Expert consensus document: Clinical and molecular diagnosis, screening and management of Beckwith-Wiedemann syndrome: an international consensus statement. Nat Rev Endocrinol 14, 229–249 (2018). 29377879 10.1038/nrendo.2017.166 PMC6022848

[21] Ruteshouser E.C., Robinson S.M. & Huff V. Wilms tumor genetics: mutations in WT1, WTX, and CTNNB1 account for only about one-third of tumors. Genes Chromosomes Cancer 47, 461–70 (2008). 18311776 10.1002/gcc.20553 PMC4332772

[22] Coorens T.H.H. Embryonal precursors of Wilms tumor. Science 366, 1247–1251 (2019). 31806814 10.1126/science.aax1323 PMC6914378

[23] Fiala E.M. 11p15.5 epimutations in children with Wilms tumor and hepatoblastoma detected in peripheral blood. Cancer 126, 3114–3121 (2020). 32320050 10.1002/cncr.32907 PMC7383476

[24] Davidson A.J., Lewis P., Przepiorski A. & Sander V. Turning mesoderm into kidney. Semin Cell Dev Biol 91, 86–93 (2019). 30172050 10.1016/j.semcdb.2018.08.016

[25] Vujanić G.M. Revised International Society of Paediatric Oncology (SIOP) working classification of renal tumors of childhood. Med Pediatr Oncol 38, 79–82 (2002). 11813170 10.1002/mpo.1276

[26] Murphy A.J. Forty-five patient-derived xenografts capture the clinical and biological heterogeneity of Wilms tumor. Nat Commun 10, 5806 (2019). 31862972 10.1038/s41467-019-13646-9 PMC6925259

[27] Sherman B.T. DAVID: a web server for functional enrichment analysis and functional annotation of gene lists (2021 update). Nucleic Acids Res (2022). 10.1093/nar/gkac194 PMC9252805 35325185

[28] Chen X. CONSERTING: integrating copy-number analysis with structural-variation detection. Nat Methods 12, 527–30 (2015). 25938371 10.1038/nmeth.3394 PMC4591043

[29] Maksimovic J., Gordon L. & Oshlack A. SWAN: Subset-quantile within array normalization for illumina infinium HumanMethylation450 BeadChips. Genome Biol 13, R44 (2012). 22703947 10.1186/gb-2012-13-6-r44 PMC3446316

[30] Aryee M.J. Minfi: a flexible and comprehensive Bioconductor package for the analysis of Infinium DNA methylation microarrays. Bioinformatics 30, 1363–9 (2014). 24478339 10.1093/bioinformatics/btu049 PMC4016708

[31] Du P. Comparison of Beta-value and M-value methods for quantifying methylation levels by microarray analysis. BMC Bioinformatics 11, 587 (2010). 21118553 10.1186/1471-2105-11-587 PMC3012676

[32] Gadd S. A Children’s Oncology Group and TARGET initiative exploring the genetic landscape of Wilms tumor. Nat Genet 49, 1487–1494 (2017). 28825729 10.1038/ng.3940 PMC5712232

[33] Mahamdallie S. Identification of new Wilms tumour predisposition genes: an exome sequencing study. Lancet Child Adolesc Health 3, 322–331 (2019). 30885698 10.1016/S2352-4642(19)30018-5 PMC6472290

[34] Zhang J. Germline Mutations in Predisposition Genes in Pediatric Cancer. N Engl J Med 373, 2336–2346 (2015). 26580448 10.1056/NEJMoa1508054 PMC4734119

[35] Landrum M.J. ClinVar: improving access to variant interpretations and supporting evidence. Nucleic Acids Res 46, D1062–D1067 (2018). 29165669 10.1093/nar/gkx1153 PMC5753237

[36] Choi Y., Sims G.E., Murphy S., Miller J.R. & Chan A.P. Predicting the functional effect of amino acid substitutions and indels. PLoS One 7, e46688 (2012). 23056405 10.1371/journal.pone.0046688 PMC3466303

[37] Choi Y. & Chan A.P. PROVEAN web server: a tool to predict the functional effect of amino acid substitutions and indels. Bioinformatics 31, 2745–7 (2015). 25851949 10.1093/bioinformatics/btv195 PMC4528627

[38] Adzhubei I.A. A method and server for predicting damaging missense mutations. Nat Methods 7, 248–9 (2010). 20354512 10.1038/nmeth0410-248 PMC2855889

[39] Song N. Persistent variations of blood DNA methylation associated with treatment exposures and risk for cardiometabolic outcomes in long-term survivors of childhood cancer in the St. Jude Lifetime Cohort. Genome Med 13, 53 (2021). 33823916 10.1186/s13073-021-00875-1 PMC8025387

[40] Huff V. Wilms’ tumours: about tumour suppressor genes, an oncogene and a chameleon gene. Nat Rev Cancer 11, 111–21 (2011). 21248786 10.1038/nrc3002 PMC4332715

[41] Armstrong A.E. A unique subset of low-risk Wilms tumors is characterized by loss of function of TRIM28 (KAP1), a gene critical in early renal development: A Children’s Oncology Group study. PLoS One 13, e0208936 (2018). 30543698 10.1371/journal.pone.0208936 PMC6292605

[42] Hol J.A. TRIM28 variants and Wilms’ tumour predisposition. J Pathol 254, 494–504 (2021). 33565090 10.1002/path.5639 PMC8252630

[43] Sy S.M., Huen M.S. & Chen J. PALB2 is an integral component of the BRCA complex required for homologous recombination repair. Proc Natl Acad Sci U S A 106, 7155–60 (2009). 19369211 10.1073/pnas.0811159106 PMC2678481

[44] Messinger Y.H. Pleuropulmonary blastoma: a report on 350 central pathology-confirmed pleuropulmonary blastoma cases by the International Pleuropulmonary Blastoma Registry. Cancer 121, 276–85 (2015). 25209242 10.1002/cncr.29032 PMC4293209

[45] Palculict T.B. Identification of germline DICER1 mutations and loss of heterozygosity in familial Wilms tumour. J Med Genet 53, 385–8 (2016). 26566882 10.1136/jmedgenet-2015-103311 PMC4866907

[46] Walz A.L. Recurrent DGCR8, DROSHA, and SIX homeodomain mutations in favorable histology Wilms tumors. Cancer Cell 27, 286–97 (2015). 25670082 10.1016/j.ccell.2015.01.003 PMC4800737

[47] Davidson A.J. & Zon L.I. Turning mesoderm into blood: the formation of hematopoietic stem cells during embryogenesis. Curr Top Dev Biol 50, 45–60 (2000). 10948449 10.1016/s0070-2153(00)50003-9

[48] Brzezinski J. Clinically and biologically relevant subgroups of Wilms tumour defined by genomic and epigenomic analyses. Br J Cancer 124, 437–446 (2021). 33012783 10.1038/s41416-020-01102-1 PMC7853092

[49] Scott R.H. Stratification of Wilms tumor by genetic and epigenetic analysis. Oncotarget 3, 327–35 (2012). 22470196 10.18632/oncotarget.468 PMC3359888

[50] Huff V. Wilms tumor genetics. Am J Med Genet 79, 260–7 (1998). 9781905 10.1002/(sici)1096-8628(19981002)79:4<260::aid-ajmg6>3.0.co;2-q

[51] Valind A. Convergent evolution of 11p allelic loss in multifocal Wilms tumors arising in WT1 mutation carriers. Pediatr Blood Cancer 65, e27301 (2018). 29968962 10.1002/pbc.27301

[52] Fukuzawa R., Heathcott R.W., More H.E. & Reeve A.E. Sequential WT1 and CTNNB1 mutations and alterations of beta-catenin localisation in intralobar nephrogenic rests and associated Wilms tumours: two case studies. J Clin Pathol 60, 1013–6 (2007). 17172473 10.1136/jcp.2006.043083 PMC1972432

[53] Maiti S., Alam R., Amos C.I. & Huff V. Frequent association of beta-catenin and WT1 mutations in Wilms tumors. Cancer Res 60, 6288–92 (2000). 11103785

[54] Royer-Pokora B. Chemotherapy and terminal skeletal muscle differentiation in WT1-mutant Wilms tumors. Cancer Med 7, 1359–1368 (2018). 29542868 10.1002/cam4.1379 PMC5911586

[55] Royer-Pokora B. Clinical relevance of mutations in the Wilms tumor suppressor 1 gene WT1 and the cadherin-associated protein beta1 gene CTNNB1 for patients with Wilms tumors: results of long-term surveillance of 71 patients from International Society of Pediatric Oncology Study 9/Society for Pediatric Oncology. Cancer 113, 1080–9 (2008). 18618575 10.1002/cncr.23672

[56] Okamoto K., Morison I.M., Taniguchi T. & Reeve A.E. Epigenetic changes at the insulin-like growth factor II/H19 locus in developing kidney is an early event in Wilms tumorigenesis. Proc Natl Acad Sci U S A 94, 5367–71 (1997). 9144243 10.1073/pnas.94.10.5367 PMC24684

[57] Harteveld C.L. Mosaic segmental uniparental isodisomy and progressive clonal selection: a common mechanism of late onset β-thalassemia major. Haematologica 98, 691–5 (2013). 22983591 10.3324/haematol.2012.065219 PMC3640111

[58] Fernandez C.V. Clinical Outcome and Biological Predictors of Relapse After Nephrectomy Only for Very Low-risk Wilms Tumor: A Report From Children’s Oncology Group AREN0532. Ann Surg 265, 835–840 (2017). 27811504 10.1097/SLA.0000000000001716 PMC5145762

[59] Kalish J.M. & Deardorff M.A. Tumor screening in Beckwith-Wiedemann syndrome-To screen or not to screen? Am J Med Genet A 170, 2261–4 (2016). 27518916 10.1002/ajmg.a.37881 PMC5930355

